# The Emerging Role of Proline in the Establishment and Functioning of Legume-*Rhizobium* Symbiosis

**DOI:** 10.3389/fpls.2022.888769

**Published:** 2022-05-27

**Authors:** Giuseppe Sabbioni, Giuseppe Forlani

**Affiliations:** Laboratory of Plant Physiology and Biochemistry, Department of Life Science and Biotechnology, University of Ferrara, Ferrara, Italy

**Keywords:** bacteroid differentiation, nitrogen fixation, nodule, proline, P5C synthetase, P5C reductase, proline dehydrogenase

## Abstract

High levels of some enzymes involved in proline synthesis and utilization were early found in soybean nodules, and rhizobial knockout mutants were shown to be defective in inducing nodulation and/or fixing nitrogen, leading to postulate that this amino acid may represent a main substrate for energy transfer from the plant to the symbiont. However, inconsistent results were reported in other species, and several studies suggested that proline metabolism may play an essential role in the legume-*Rhizobium* symbiosis only under stress. Different mechanisms have been hypothesized to explain the beneficial effects of proline on nodule formation and bacteroid differentiation, yet none of them has been conclusively proven. Here, we summarize these findings, with special emphasis on the occurrence of a legume-specific isoform of δ^1^-pyrroline-5-carboxylate synthetase, the enzyme that catalyses the rate-limiting step in proline synthesis. Data are discussed in view of recent results connecting the regulation of both, the onset of nodulation and proline metabolism, to the redox status of the cell. Full comprehension of these aspects could open new perspectives to improve the adaptation of legumes to environmental stress.

## Proline Synthesis in Legume Nodules as a Possible Mechanism for Energy Transfer to the Bacteroids

**Figure 1:**
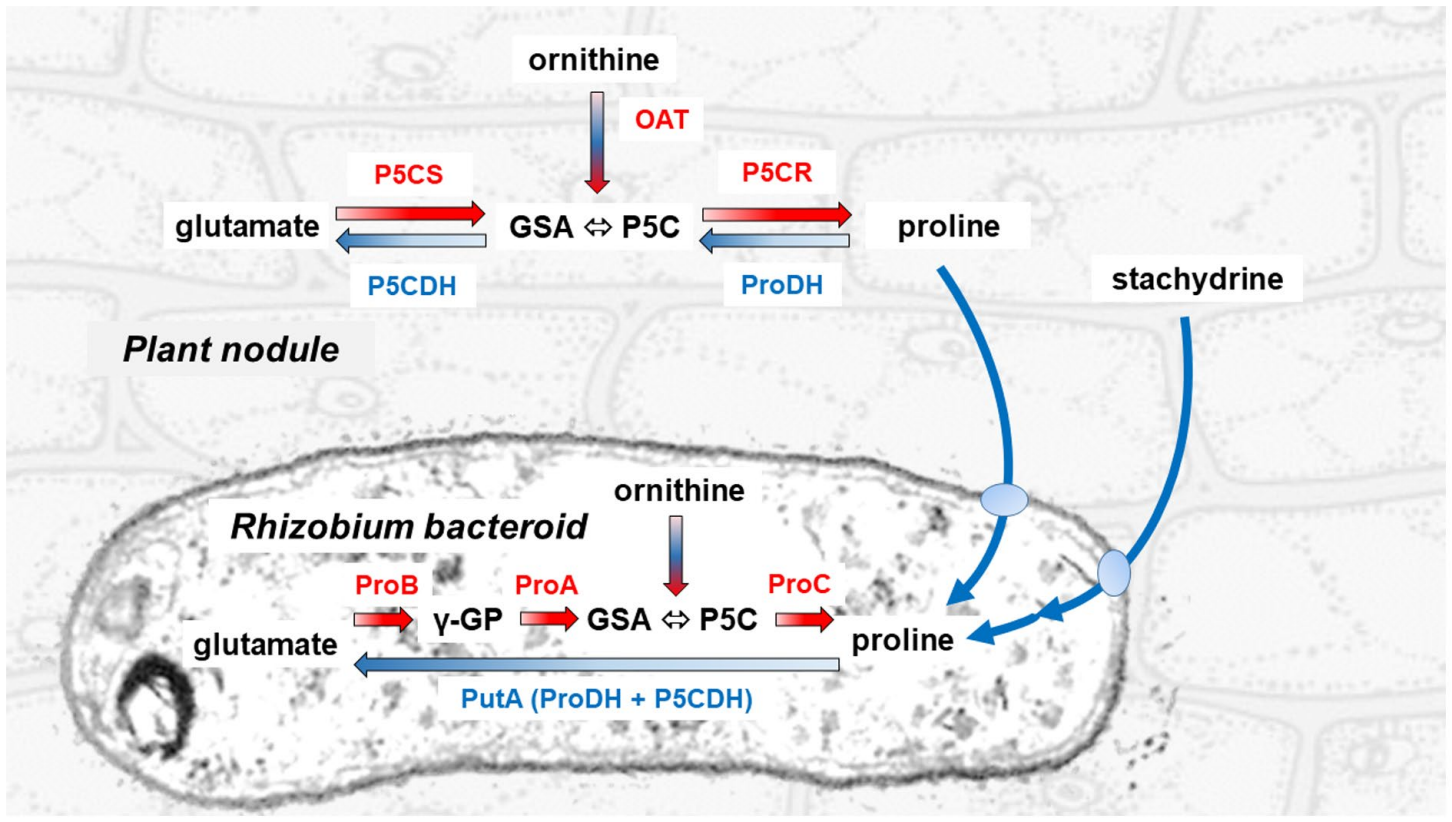
Pathways for proline synthesis and catabolism in the legume plant and the rhizobial symbiont. The synthesis of proline proceeds in the cytoplasm of the plant cell through the sequential action of a bifunctional P5C synthetase (P5CS) that reduces glutamate to glutamate semialdehyde (GSA, which spontaneously cyclizes to P5C), and of a P5C reductase (P5CR) that reduces the latter to proline. In the bacteroid, the same initial reaction is catalyzed by two distinct enzymes, a γ-glutamyl-kinase (ProB) and a glutamyl-phosphate reductase (ProA). Conversely, proline is oxidized back to glutamate in the plant mitochondrion through two steps, catalyzed by a proline dehydrogenase (ProDH) that feeds electrons directly to the respiratory chain, and a P5C dehydrogenase (P5CDH). In the bacterial symbiont, these reactions are mediated by a single proline oxidase (PutA) bearing both catalytic domains of ProDH and P5CDH. GSA/P5C is generated also during arginine catabolism by means of an ornithine-δ-aminostransferase (OAT). Utilization of stachydrine, also known as proline betaine, a compatible osmolyte that is accumulated in several legumes under stress, proceeds in the rhizobium by means of some proline-releasing enzymatic activities, whose genes are grouped on the Sym plasmid (Goldmann et al., 1994).

Legume-*Rhizobium* symbiosis is a mutually beneficial association between higher plants and microbes, during which biological nitrogen fixation occurs in the nodule, a specialized accessory legume organ usually formed on roots. In mature nodules, rhizobia differentiate into bacteroids and convert N_2_ into ammonia, essential for plant growth. In return, bacteria obtain carbon and energy sources from the plant (Wang et al., 2018). For nitrogen transport, legumes of tropical origin use ureides that derive from purine oxidation. Sustained purine production requires increased NADP^+^ availability, which might be obtained through increased proline biosynthesis. In the pioneeristic work of Kohl et al. (1988), the levels, properties, and subcellular location of δ^1^-pyrroline-5-carboxylate (P5C) reductase (EC 1.5.1.2, P5CR) and proline dehydrogenase (EC 1.5.5.2, ProDH), the enzymes that catalyze the last step in proline synthesis and the first step in proline catabolism, respectively (Figure 1), were investigated in nodules of soybean (*Glycine max*), an ureide exporting species. P5CR levels were found 20-fold higher in nodule plant cytosol than in leaf extracts, and about 4-fold higher than in nodules of pea, an amide-exporting species. NADH-dependent P5CR activity was insensitive to proline up to 4 mM, whereas it was inhibited by 60% in the presence of 1 mM NADP^+^. These findings led these authors to hypothesize a main role for P5CR in regenerating NADP^+^ for the oxidative pentose phosphate pathway (OPPP) to support purine biosynthesis.

High levels of ProDH were also detected in soybean nodules, only 2% of which were present in mitochondria-enriched fractions. Therefore, it was hypothesized that at least part of the proline synthesized in the plant is transferred to the bacteroids, where it is used as an energy source. A susceptibility to feed-back inhibition by proline only at concentrations exceeding 5 mM, and a reduced activity in the presence of NADP^+^ concentrations in the range 10^−5^–10^−3^ M, were later shown as common traits of plant P5CRs (Giberti et al., 2014; Forlani et al., 2015; Ruszkowski et al., 2015). Moreover, when P5CR and OPPP activity levels were measured in extracts from nodules of eight different legumes, no differences were found between ureide and amide-exporting species, and the initial hypothesis of a role of P5CR in sustaining ureide synthesis was not supported (Kohl et al., 1990). High activities of both P5CR and OPPP were found anyway in nodules of all species, and substantial levels of ProDH were also detected. ProDH activity in bacteroids was high enough during the soybean growing season to supply a significant fraction of the energy required for nitrogen fixation, and even higher levels were found in other legumes, such as *Phaseolus vulgaris* (Kohl et al., 1990). Interestingly, mean ProDH activity levels in ureide-exporting legumes were about 3-fold higher than in amide-exporting species. Being ProDH subjected to a strict control at the transcriptional level by the intracellular concentration of free proline (Liu et al., 2017), this could imply that lower amounts of proline are transferred to the symbionts in legumes of temperate climate origin. However, this aspect was not further considered.

When the properties of P5CR from soybean and *Bradyrhizobium japonicum* were compared, the rhizobial enzyme showed a 6-fold higher affinity for P5C and a 2–3-fold higher specific activity (Chilson et al., 1992). An active proline synthesis in bacteroids would be inconsistent with its role as a possible energy source, leading to a wasteful futile cycle. Contrary to the plant enzyme, *B. japonicum* P5CR was insensitive to NADP^+^ at 0.29 mM, and 50%-inhibited by 20 mM proline, with a K_I_ value for proline (1.8 mM) similar to the concentration of the amino acid in bacteroids (Chilson et al., 1992). However, these features were evaluated using only NADH as the electron donor, while increasing evidence has been found supporting a preferential use *in vivo* of NADPH in both higher plants (Giberti et al., 2014) and bacteria (Petrollino and Forlani, 2012). The occurrence of post-translational regulative mechanisms modulating P5CR in bacteroids, which may allow utilization of exogenous proline as an energy source, therefore, cannot be ruled out.

## Effect of Disruption of the Bacterial Genes Coding for P5CR and ProDH on the Establishment of Legume-*Rhizobium* Symbiosis

To shed more light on the role of proline metabolism in bacteroids, some mutants impaired in either proline synthesis or utilization were characterized. A *B. japonicum* strain, in which the gene coding for P5CR had been disrupted, was found to be a strict proline auxotroph (King et al., 2000). Therefore, in this species, proline synthesis cannot be accomplished through alternative biosynthetic routes described in other bacteria (Fichman et al., 2015). This strain was unable to elicit nitrogen fixing nodules on soybean: only undeveloped nodules were evident that contained few viable bacteria, without detectable amounts of leghemoglobin (King et al., 2000). These results clearly pointed out that during the establishment of the symbiosis the plant is unable to provide the symbiont with amounts of proline high enough to satisfy its auxotrophic requirement. However, the possibility that a significant proline transfer to the bacteroids takes place at a later stage, when the nodule has completely developed, cannot be excluded.

A *Sinorhizobium meliloti* p5cr strain, in which a gene for ornithine cyclodeaminase had also been disrupted to prevent proline synthesis from arginine, showed a different phenotype depending on the host plant. On white sweet clover (*Melilotus alba*), nodulation was slightly delayed, but the final number of nodules *per* plant was similar to that found with the wild-type strain. However, nodules failed to fix nitrogen and showed the presence of few differentiated bacteroids compared to wild-type-filled nodules. On alfalfa (*Medicago sativa*), the p5cr strain was on the contrary able to induce fully functional nodules, although a decrease in nodule mass and, consequently, in nitrogen fixing rate was found (Dicenzo et al., 2015). The differential effect was interpreted as due to stachydrine (proline betaine) production in alfalfa, which can be converted to proline by *S. meliloti*. Whatever the exact mechanism, these results supported a requirement for proline prototrophy during bacteroid differentiation. Conversely, these data also suggested that -once bacteroid differentiation occurred- active proline synthesis in the bacterial symbiont is dispensable.

Concerning proline catabolism, a cowpea *Rhizobium* strain was found able to use the amino acid as the only carbon source. This notwithstanding, neither bacteroids isolated from *Vigna unguiculata* were capable of proline uptake, nor detectable proline levels were found in snakebean nodule cytosol (Glenn et al., 1991). When a group of *Rhizobium leguminosarum* mutants impaired in either proline synthesis or catabolism were investigated, mutations had a negligible impact on nitrogenase activity, and proline levels in the nodule plant cytosol and in bacteroids did not support proline transfer between the symbionts (Chien et al., 1991). Following insertional mutagenesis and *ProDH* disruption, a strain of *S. meliloti* was shown unable to use either proline or ornithine as the sole nitrogen source. Once again, when the nodulation of this mutant on alfalfa was considered, no obvious defects were found, and the nitrogen fixation rate was unaffected, although full nodulation was slightly delayed and the number of nodule *per* plant reduced. More pronounced effects were evident in competition assays: after 1:1 inoculation with the wild-type parental, only 6% of nodules were found occupied by the mutant strain (Jiménez-Zurdo et al., 1995). Similar results were obtained with pigeonpea plants colonized by *Rhizobium* sp. (*Cajanus*), although in this case the prodh strain was unable to induce functional nodules (Sharma and Yadav, 2012). Besides suggesting the occurrence of a significant variability among strains as to this point, these results demonstrated not only that proline is not *the sole* energy-yielding compound transferred to the bacteroid, but also that proline catabolism in rhizobia may influence the efficiency of nitrogen fixation. Consistently, another gene involved in the catabolism of stachydrine, whose utilization proceeds through proline, was found to be required for efficient nodulation (Goldmann et al., 1994).

## Protective Role of Proline in Nodules Under Hyperosmotic Stress

Because of the well-established role of proline (Furlan et al., 2020) and stachydrine (Trinchant et al., 2004) in the legume response to osmotic stress, numerous studies focused on the possibility that proline transfer from the plant host to the rhizobial symbiont may be functional to preserve nodule efficiency under drought or excess salt. Osmotic stress impairs both, the photosynthesis and the photosynthate transport to the nodules, thereby decreasing nitrogen fixation up to 90%. In soybean, proline levels in nodules from osmotically stressed plants were found 3–4-fold higher than in unstressed controls, despite a 2–4-fold increase of ProDH activity (Kohl et al., 1991). The exogenous supply of proline to intact soybean plants increased its intracellular concentration in bacteroids from 0.45 to 3.6 mM, causing in turn an increase of ProDH activity that was highly correlated with the resulting rate of acetylene reduction. Similar effects were also induced by exogenous succinate. However, proline application did not rescue stemgirdled plants from loss of nitrogenase activity, whereas succinate application did, suggesting a phloematic transport of proline (Zhu et al., 1992). Such evidence led these authors to hypothesize that, although dicarboxylates are the main compounds used for energy transfer from the plant to the bacteroids, proline does contribute, and may play a key role under environmental stress and the subsequent recovery (Kohl et al., 1994). Consistently, when nitrogenase was measured in bacteroids isolated from soybean, enzyme activity supported by proline was 8-fold higher in bacteroids from drought-stressed nodules than in bacteroids from controls, while no effect was found with the succinate-dependent activity. Results also showed an increase in the rate of proline uptake relative to a minor decrease in malate uptake into stressed symbiosomes (Pedersen et al., 1996).

To evaluate whether this may have an agronomic significance, soybean plants were inoculated with either a wild-type or a prodh strain of *B. japonicum*, and seed production was measured following the imposition of water stress conditions. When the stress was mild, plants inoculated with the mutant strain suffered twice the percentage yield decrease than plants inoculated with bacteria able to catabolize proline, whereas no difference was found under severe drought (Straub et al., 1997). The positive impact on nitrogen fixation (and on seed yield in turn) of proline catabolism under mild water stress was further confirmed on soybean plants grown in the absence of alternative sources of nitrogen (Curtis et al., 2004). However, when *ProDH* expression was followed during the symbiotic interaction between *Rhizobium meliloti* and alfalfa plants, transcription was induced by root exudates and during nodule formation, but not in differentiated nitrogen-fixing bacteroids. In this case, a prodh strain showed strongly reduced colonization efficiency (Jiménez-Zurdo et al., 1997).

Conversely, stress-induced production of proline and stachydrine in the plant host was found to be paralleled by an increase of proline levels in bacteroids. Exposure of nodulated alfalfa plants to 0.2 M NaCl for 4 weeks strongly reduced stachydrine turnover, leading to a 4–10-fold increase of its levels in plant tissues. As a consequence, stachydrine and proline concentrations in salt-stressed bacteroids reached 7.4 and 11.8 mM, respectively, up to 15-fold the levels in untreated controls. Ultrastructural analysis showed a large peribacteroid space in salt-stressed nodules, suggesting an increased turgor pressure inside the symbiosomes (Trinchant et al., 2004). A possible and consequent induction of proline catabolism in bacteroids was not investigated.

Transgenic *Medicago truncatula* plants overexpressing P5C synthetase (P5CS), the bifunctional enzyme catalyzing the first and limiting step in proline synthesis (Figure 1), showed higher proline levels in roots, leaves, and nodules. When subjected to osmotic stress, nitrogen fixation by *S. meliloti* was significantly less affected than in untransformed plants. A strong *ProDH* upregulation was found in nodules of both wild-type and transgenic salt-stressed plants (Verdoy et al., 2006). These authors also hypothesized a different mechanism by which increased proline availability may benefit nodule functioning, i.e., through enhanced production of proline-rich proteins, which may provide mechanical support for the cell under stress, and whose transcripts accumulate in bean nodules as a response to drought and excess salt (Verdoy et al., 2004). Analogous results were reported in the case of *M. truncatula* plants constitutively expressing a gene for arginine decarboxylase, an enzyme involved in polyamine synthesis (Hidalgo-Castellanos et al., 2019). Following the exposure to salt stress, transgenic plants showed higher nitrogenase activity and nodule number and biomass. Interestingly, proline was found to increase in nodules of transformed plants but not in untransformed controls, which accumulated glutamate and γ-aminobutyric acid instead (Hidalgo-Castellanos et al., 2019).

## High Proline Levels in Legumes as Determined by a Specific Form of P5CS

When a salt-resistant and a salt-sensitive alfalfa cultivar were inoculated with a salt-sensitive and a salt-resistant *S. meliloti* strain in all four possible combinations, amino acid composition of nodules under salt stress was found to depend mainly on the rhizobial strain. In all plants, total free amino acid concentration was much higher in nodules than in roots and leaves, where NaCl treatment was the major driving factor for amino acid accumulation. In both salt-stressed and unstressed nodules, the salt-tolerant rhizobial strain caused a significant increase of most amino acids of the glutamate family, comprising proline, whereas the salt-sensitive strain triggered a remarkable increase of amino acids of the aspartate family (Bertrand et al., 2016). Since amino acids of the glutamate family play a main role in nitrogen assimilation, osmoprotection, and energy conservation (Okumoto et al., 2016), the superior performance under stress of the symbiosis between a salt-tolerant plant and a salt-tolerant symbiont may depend, at least in part, on their specific accumulation.

**Figure 2:**
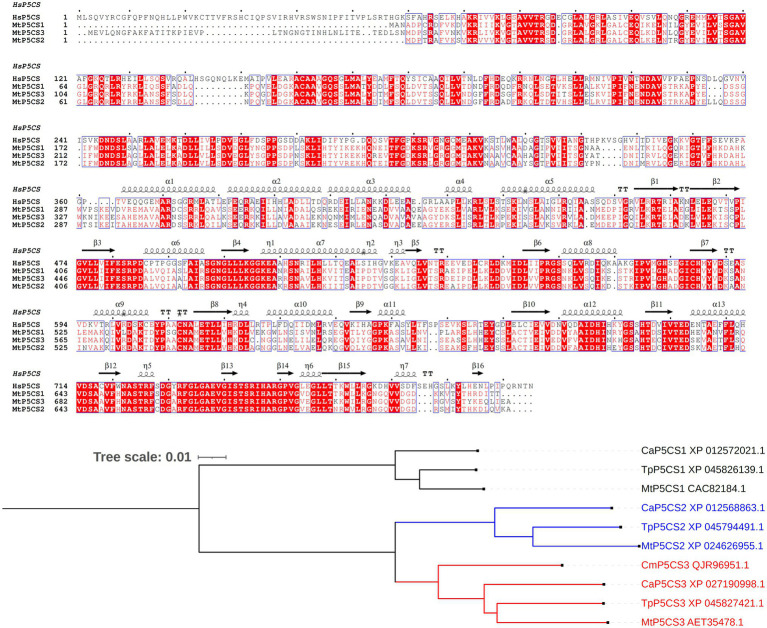
Legume-specific isozymes of δ^1^-pyrroline-5-carboxylate synthetase. **Upper panel**: Multiple sequence alignment of *Medicago truncatula* P5CS isoforms. The three proteins described in barrel clover (Kim and Nam, 2013) and the human enzyme (X94453.1) have been aligned using Clustal Omega (Madeira et al., 2019) and Espript 3.0 (Robert and Gouet, 2014). Sequence identities are emphasized in red and similarities are highlighted as red letters. The corresponding secondary structure of *Homo sapiens* P5CS, the only enzyme form whose architecture has been partially resolved (PDB id 2H5G), is shown on the top. Helices appear as scribble, β-strands as arrows. **Lower panel**: Occurrence of a third P5CS isozyme in other legumes. The sequence of the 43-aa extra amino-terminal segment of *Mt*P5CS3 was used as a query for a sequence similarity search using BLAST (https://blast.ncbi.nlm.nih.gov/Blast.cgi). No significant similarity was found other than in legumes. A few incomplete or redundant sequences were discarded, resulting in three sequences from *Trifolium pratense*, *Cicer arietinum*, and *Caragana microphylla*. These sequences were aligned with the other two *canonical* P5CS isoforms, and used for maximum likelihood tree reconstruction using Clustal Omega with default parameters. Tree visualization was obtained using iTol, version 6.5.2 (Letunic and Bork, 2021). For *C. microphylla*, P5CS1 and P5CS2 sequences were not available.

A major achievement toward a better understanding of the role of proline in the legume-rhizobium symbiosis was made with the discovery of a legume-specific form of P5CS (Kim and Nam, 2013). The occurrence of two P5CS isogenes has in fact been reported in several plant species, and phylogenetic analyses demonstrated that gene duplication occurred independently in several taxonomic groups (Turchetto-Zolet et al., 2009; Rai and Penna, 2013). These paralogs, named P5CS1 and P5CS2, show non-overlapping roles, with varying temporal and spatial expression patterns. Overall, P5CS1 has been identified as the major contributor to stress-induced proline accumulation, whereas P5CS2 plays a pivotal role in embryo development and growth (Fichman et al., 2015). In *M. truncatula*, a third isozyme was found, showing an extra amino-terminal segment (Figure 2). Under salt excess and drought, *MtP5CS3* is expressed in shoots and nodulating roots at levels higher than the other isogenes, and a loss-of-function p5cs3 mutant accumulated less proline, formed fewer nodules, and showed lower nitrogen fixation rates than the wild-type (Kim and Nam, 2013).

The occurrence of a third P5CS isoform has not been reported in other legumes. However, a homology search based on the deduced sequence of the extra 43 amino acids segment of *Mt*P5CS3 showed the presence of a similar system in other species, such as *Trifolium pratense*, *Cicer arietinum*, and *Caragana microphylla*. A maximum likelihood tree reconstruction suggests that this legume-specific form derives from a duplication of *P5CS2* (Figure 2). It would be interesting to assess whether *Mt*P5CS3 is also subjected to peculiar post-translational regulation mechanisms, such as a lack of feed-back inhibition by proline, yet this aspect has been poorly investigated to date (Sabbioni et al., 2021). Anyway, its emergence seems to imply that a higher demand for proline is connected with symbiotic nitrogen fixation, and further strengthens the possibility that high proline levels are needed for an optimal functioning of the nodule under stress.

## Possible Roles for Proline in the Establishment of the Legume-*Rhizobium* Symbiosis Besides as an Energy Source

Some inconsistent results in legumes other than soybean questioned the possibility that proline may represent an essential compound for energy transfer from the plant to the rhizobial symbiont. Other roles have been hypothesized to explain the beneficial effect of an active proline metabolism on nodule formation and nitrogen fixation. Among them, the attainment of high protein synthesis rate during bacteroid differentiation, the synthesis of proline-rich proteins to stabilize the cell wall under osmotic stress conditions, an osmotic stabilization of the nodule environment under (mild) stress, or the provision of a high-yield energy source useful as microenvironmental stress substrate. A preferential use of proline as high-yield energy source has found confirmation in some other systems, ranging from inflammation and tumorigenesis (Phang et al., 2008) to the earliest or most expensive stages of insect flight (Bertazzini et al., 2010).

Another intriguing possibility comes from recent advances in our comprehension of the connection between both, the nodulation process and proline metabolism, and the redox status of the cell. Nodule metabolism continuously generates reactive oxygen (ROS) and nitrogen (RNS) species, which during evolution have been recruited as versatile signaling molecules. An increasing body of evidence shows that the balance between such reactive species and antioxidants systems regulates the onset of symbiosis and nodule development through controlling the redox status in legume nodules (Damiani et al., 2016; Matamoros and Becana, 2020). Although the possibility that proline may act as ROS scavenger has not been confirmed (Forlani et al., 2019), several recent studies showed that the interconversion of glutamate and proline may regulate the redox balance and the energy status of the cell in connection with other redox shuttles (Zheng et al., 2021). Accordingly, most enzymes in proline metabolism influence, and are regulated by, the intracellular NAD(P)^+^/NAD(P)H ratio (Giberti et al., 2014; Sabbioni et al., 2021). Therefore, active proline metabolism in nodules could be involved in generating ROS and/or maintaining redox levels required for a proper establishment of the symbiosis.

## Concluding Remarks

High levels of proline synthesis and utilization are found during nodulation and bacteroid differentiation, whose perturbation leads to reduced nodule mass and number, and to decreased nitrogen fixation. Although the exact mechanism by which proline metabolism benefits the establishment of the legume-*Rhizobium* symbiosis is still far to be fully understood, it seems to play a main role in maintaining nodule efficiency under stress. Shedding light on the molecular basis of these effects will provide a reliable tool to handle nodule activity and, hopefully, improve the adaptation of legumes to environmental stress, a main goal in view of the ongoing climate changes.

## Author Contributions

All authors listed have made a substantial, direct, and intellectual contribution to the work and approved it for publication.

## Funding

This work was supported in part by the University of Ferrara in the frame of the project FAR 2020.

## Conflict of Interest

The authors declare that the research was conducted in the absence of any commercial or financial relationships that could be construed as a potential conflict of interest.

## Publisher’s Note

All claims expressed in this article are solely those of the authors and do not necessarily represent those of their affiliated organizations, or those of the publisher, the editors and the reviewers. Any product that may be evaluated in this article, or claim that may be made by its manufacturer, is not guaranteed or endorsed by the publisher.
